# Antenatal and delivery practices and neonatal mortality amongst women with institutional and non-institutional deliveries in rural Zimbabwe: observational data from a cluster randomized trial

**DOI:** 10.1186/s12884-022-05282-x

**Published:** 2022-12-30

**Authors:** Christie Noble, Ciaran Mooney, Rachel Makasi, Robert Ntozini, Florence D. Majo, James A. Church, Naume V. Tavengwa, Andrew J. Prendergast, Jean H. Humphrey, for the Sanitation Hygiene Infant Nutrition Efficacy (SHINE) Trial Team, Andrew D. Jones, Amee Manges, Goldberg Mangwadu, John A. Maluccio, Mduduzi N. N. Mbuya, Lawrence H. Moulton, Rebecca J. Stoltzfus, James M. Tielsch, Laura E. Smith, Cynthia Chasokela, Ancikaria Chigumira, William Heylar, Preston Hwena, George Kembo, Batsirai Mutasa, Kuda Mutasa, Philippa Rambanepasi, Virginia Sauramba, Franne Van Der Keilen, Chipo Zambezi, Dzivaidzo Chidhanguro, Dorcas Chigodora, Joseph F. Chipanga, Grace Gerema, Tawanda Magara, Mandava Mandava, Tafadzwa Mavhudzi, Clever Mazhanga, Grace Muzaradope, Marian T. Mwapaura, Simon Phiri, Alice Tengende, Cynthia Banda, Bernard Chasekwa, Leah Chidamba, Theodore Chidawanyika, Elisha Chikwindi, Lovemore K. Chingaona, Courage K. Chiorera, Adlight Dandadzi, Margaret Govha, Hlanai Gumbo, Karen T. Gwanzura, Sarudzai Kasaru, Alois M. Matsika, Diana Maunze, Exevia Mazarura, Eddington Mpofu, Johnson Mushonga, Tafadzwa E. Mushore, Tracey Muzira, Netsai Nembaware, Sibongile Nkiwane, Penias Nyamwino, Sandra D. Rukobo, Thompson Runodamoto, Shepherd Seremwe, Pururudzai Simango, Joice Tome, Blessing Tsenesa, Umali Amadu, Beauty Bangira, Daniel Chiveza, Priscilla Hove, Horaiti A. Jombe, Didymus Kujenga, Lenin Madhuyu, Prince Mandina-Makoni, Naume Maramba, Betty Maregere, Ellen Marumani, Elisha Masakadze, Phathisiwe Mazula, Caroline Munyanyi, Grace Musanhu, Raymond C. Mushanawani, Sibongile Mutsando, Felicia Nazare, Moses Nyarambi, Wellington Nzuda, Trylife Sigauke, Monica Solomon, Tendai Tavengwa, Farisai Biri, Misheck Chafanza, Cloud Chaitezvi, Tsundukani Chauke, Collen Chidzomba, Tawanda Dadirai, Clemence Fundira, Athanasios C. Gambiza, Tatenda Godzongere, Maria Kuona, Tariro Mafuratidze, Idah Mapurisa, Tsitsi Mashedze, Nokuthula Moyo, Charles Musariri, Matambudzo Mushambadope, Tawanda R. Mutsonziwa, Augustine Muzondo, Rudo Mwareka, Juleika Nyamupfukudza, Baven Saidi, Tambudzai Sakuhwehwe, Gerald Sikalima, Jenneth Tembe, Tapiwanashe E. Chekera, Owen Chihombe, Muchaneta Chikombingo, Tichaona Chirinda, Admire Chivizhe, Ratidzai Hove, Rudo Kufa, Tatenda F. Machikopa, Wilbert Mandaza, Liberty Mandongwe, Farirai Manhiyo, Emmanuel Manyaga, Peter Mapuranga, Farai S. Matimba, Patience Matonhodze, Sarah Mhuri, Joice Mike, Bekezela Ncube, Walter T. S. Nderecha, Munyaradzi Noah, Charles Nyamadzawo, Jonathan Penda, Asinje Saidi, Sarudzai Shonhayi, Clemence Simon, Monica Tichagwa, Rachael Chamakono, Annie Chauke, Andrew F. Gatsi, Blessing Hwena, Hillary Jawi, Benjamin Kaisa, Sithembile Kamutanho, Tapiwa Kaswa, Paradhi Kayeruza, Juliet Lunga, Nomatter Magogo, Daniel Manyeruke, Patricia Mazani, Fungai Mhuriyengwe, Farisai Mlambo, Stephen Moyo, Tawanda Mpofu, Mishelle Mugava, Yvonne Mukungwa, Fungai Muroyiwa, Eddington Mushonga, Selestino Nyekete, Tendai Rinashe, Kundai Sibanda, Milton Chemhuru, Jeffrey Chikunya, Vimbai F. Chikwavaire, Charity Chikwiriro, Anderson Chimusoro, Jotam Chinyama, Gerald Gwinji, Nokuthula Hoko-Sibanda, Rutendo Kandawasvika, Tendai Madzimure, Brian Maponga, Antonella Mapuranga, Joana Marembo, Luckmore Matsunge, Simbarashe Maunga, Mary Muchekeza, Monica Muti, Marvin Nyamana, Efa Azhuda, Urayai Bhoroma, Ailleen Biriyadi, Elizabeth Chafota, Angelline Chakwizira, Agness Chamhamiwa, Tavengwa Champion, Stella Chazuza, Beauty Chikwira, Chengeto Chingozho, Abigail Chitabwa, Annamary Dhurumba, Albert Furidzirai, Andrew Gandanga, Chipo Gukuta, Beauty Macheche, Bongani Marihwi, Barbara Masike, Eunice Mutangandura, Beatrice Mutodza, Angeline Mutsindikwa, Alice Mwale, Rebecca Ndhlovu, Norah Nduna, Cathrine Nyamandi, Elias Ruvata, Babra Sithole, Rofina Urayai, Bigboy Vengesa, Micheal Zorounye, Memory Bamule, Michael Bande, Kumbirai Chahuruva, Lilian Chidumba, Zvisinei Chigove, Kefas Chiguri, Susan Chikuni, Ruvarashe Chikwanda, Tarisai Chimbi, Micheal Chingozho, Olinia Chinhamo, Regina Chinokuramba, Chiratidzo Chinyoka, Xaviour Chipenzi, Raviro Chipute, Godfrey Chiribhani, Mary Chitsinga, Charles Chiwanga, Anamaria Chiza, Faith Chombe, Memory Denhere, Ephania Dhamba, Miriam Dhamba, Joyas Dube, Florence Dzimbanhete, Godfrey Dzingai, Sikhutele Fusira, Major Gonese, Johnson Gota, Kresencia Gumure, Phinias Gwaidza, Margret Gwangwava, Winnet Gwara, Melania Gwauya, Maidei Gwiba, Joyce Hamauswa, Sarah Hlasera, Eustina Hlukani, Joseph Hotera, Lovemore Jakwa, Gilbert Jangara, Micheal Janyure, Christopher Jari, Duvai Juru, Tabeth Kapuma, Paschalina Konzai, Moly Mabhodha, Susan Maburutse, Chipo Macheka, Tawanda Machigaya, Florence Machingauta, Eucaria Machokoto, Evelyn Madhumba, Learnard Madziise, Clipps Madziva, Mavis Madzivire, Mistake Mafukise, Marceline Maganga, Senzeni Maganga, Emmanuel Mageja, Miriam Mahanya, Evelyn Mahaso, Sanelisiwe Mahleka, Pauline Makanhiwa, Mavis Makarudze, Constant Makeche, Nickson Makopa, Ranganai Makumbe, Mascline Mandire, Eunice Mandiyanike, Eunice Mangena, Farai Mangiro, Alice Mangwadu, Tambudzai Mangwengwe, Juliet Manhidza, Farai Manhovo, Irene Manono, Shylet Mapako, Evangelista Mapfumo, Timothy Mapfumo, Jane Mapuka, Douglas Masama, Getrude Masenge, Margreth Mashasha, Veronica Mashivire, Moses Matunhu, Pazvichaenda Mavhoro, Godfrey Mawuka, Ireen Mazango, Netsai Mazhata, David Mazuva, Mary Mazuva, Filomina Mbinda, John Mborera, Upenyu Mfiri, Florence Mhandu, Chrispen Mhike, Tambudzai Mhike, Artwell Mhuka, Judith Midzi, Siqondeni Moyo, Michael Mpundu, Nicholas Msekiwa Msindo, Dominic Msindo, Choice Mtisi, Gladys Muchemwa, Nyadziso Mujere, Ellison Mukaro, Kilvera Muketiwa, Silvia Mungoi, Esline Munzava, Rosewita Muoki, Harugumi Mupura, Evelyn Murerwa, Clarieta Murisi, Letwin Muroyiwa, Musara Muruvi, Nelson Musemwa, Christina Mushure, Judith Mutero, Philipa Mutero, Patrick Mutumbu, Cleopatra Mutya, Lucia Muzanango, Martin Muzembi, Dorcus Muzungunye, Valeliah Mwazha, Thembeni Ncube, Takunda Ndava, Nomvuyo Ndlovu, Pauline Nehowa, Dorothy Ngara, Leonard Nguruve, Petronella Nhigo, Samukeliso Nkiwane, Luckson Nyanyai, Judith Nzombe, Evelyn Office, Beatrice Paul, Shambadzirai Pavari, Sylvia Ranganai, Stella Ratisai, Martha Rugara, Peter Rusere, Joyce Sakala, Prosper Sango, Sibancengani Shava, Margaret Shekede, Cornellious Shizha, Tedla Sibanda, Neria Tapambwa, John Tembo, Netsai Tinago, Violet Tinago, Theresa Toindepi, John Tovigepi, Modesta Tuhwe, Kundai Tumbo, Tinashe Zaranyika, Tongai Zaru, Kamurayi Zimidzi, Matilda Zindo, Maria Zindonda, Nyaradzai Zinhumwe, Loveness Zishiri, Emerly Ziyambi, James Zvinowanda, Ekenia Bepete, Christine Chiwira, Naume Chuma, Abiegirl Fari, Samson Gavi, Violet Gunha, Fadzai Hakunandava, Constance Huku, Given Hungwe, Grace Maduke, Elliot Manyewe, Tecla Mapfumo, Innocent Marufu, Chenesai Mashiri, Shellie Mazenge, Euphrasia Mbinda, Abigail Mhuri, Charity Muguti, Lucy Munemo, Loveness Musindo, Laina Ngada, Dambudzo Nyembe, Rachel Taruvinga, Emma Tobaiwa, Selina Banda, Jesca Chaipa, Patricia Chakaza, Macdonald Chandigere, Annie Changunduma, Chenesai Chibi, Otilia Chidyagwai, Elika Chidza, Nora Chigatse, Lennard Chikoto, Vongai Chingware, Jaison Chinhamo, Marko Chinhoro, Answer Chiripamberi, Esther Chitavati, Rita Chitiga, Nancy Chivanga, Tracy Chivese, Flora Chizema, Sinikiwe Dera, Annacolleta Dhliwayo, Pauline Dhononga, Ennia Dimingo, Memory Dziyani, Tecla Fambi, Lylian Gambagamba, Sikangela Gandiyari, Charity Gomo, Sarah Gore, Jullin Gundani, Rosemary Gundani, Lazarus Gwarima, Cathrine Gwaringa, Samuel Gwenya, Rebecca Hamilton, Agnes Hlabano, Ennie Hofisi, Florence Hofisi, Stanley Hungwe, Sharai Hwacha, Aquiiline Hwara, Ruth Jogwe, Atanus Kanikani, Lydia Kuchicha, Mitshel Kutsira, Kumbulani Kuziyamisa, Mercy Kuziyamisa, Benjamin Kwangware, Portia Lozani, Joseph Mabuto, Vimbai Mabuto, Loveness Mabvurwa, Rebecca Machacha, Cresenzia Machaya, Roswitha Madembo, Susan Madya, Sheneterai Madzingira, Lloyd Mafa, Fungai Mafuta, Jane Mafuta, Alfred Mahara, Sarudzai Mahonye, Admire Maisva, Admire Makara, Margreth Makover, Ennie Mambongo, Murenga Mambure, Edith Mandizvidza, Gladys Mangena, Elliot Manjengwa, Julius Manomano, Maria Mapfumo, Alice Mapfurire, Letwin Maphosa, Jester Mapundo, Dorcas Mare, Farai Marecha, Selina Marecha, Christine Mashiri, Medina Masiya, Thembinkosi Masuku, Priviledge Masvimbo, Saliwe Matambo, Getrude Matarise, Loveness Matinanga, John Matizanadzo, Margret Maunganidze, Belinda Mawere, Chipiwa Mawire, Yulliana Mazvanya, Maudy Mbasera, Magret Mbono, Cynthia Mhakayakora, Nompumelelo Mhlanga, Bester Mhosva, Nomuhle Moyo, Over Moyo, Robert Moyo, Charity Mpakami, Rudo Mpedzisi, Elizabeth Mpofu, Estery Mpofu, Mavis Mtetwa, Juliet Muchakachi, Tsitsi Mudadada, Kudakwashe Mudzingwa, Mejury Mugwira, Tarsisio Mukarati, Anna Munana, Juliet Munazo, Otilia Munyeki, Patience Mupfeka, Gashirai Murangandi, Maria Muranganwa, Josphine Murenjekwa, Nothando Muringo, Tichafara Mushaninga, Florence Mutaja, Dorah Mutanha, Peregia Mutemeri, Beauty Mutero, Edina Muteya, Sophia Muvembi, Tandiwe Muzenda, Agnes Mwenjota, Sithembisiwe Ncube, Tendai Ndabambi, Nomsa Ndava, Elija Ndlovu, Eveln Nene, Enniah Ngazimbi, Atalia Ngwalati, Tafirenyika Nyama, Agnes Nzembe, Eunica Pabwaungana, Sekai Phiri, Ruwiza Pukuta, Melody Rambanapasi, Tambudzai Rera, Violet Samanga, Sinanzeni Shirichena, Chipiwa Shoko, More Shonhe, Cathrine Shuro, Juliah Sibanda, Edna Sibangani, Nikisi Sibangani, Norman Sibindi, Mercy Sitotombe, Pearson Siwawa, Magret Tagwirei, Pretty Taruvinga, Antony Tavagwisa, Esther Tete, Yeukai Tete, Elliot Thandiwe, Amonilla Tibugari, Stella Timothy, Rumbidzai Tongogara, Lancy Tshuma, Mirirayi Tsikira, Constance Tumba, Rumbidzayi Watinaye, Ethel Zhiradzango, Esther Zimunya, Leanmary Zinengwa, Magret Ziupfu, Job Ziyambe

**Affiliations:** 1 Blizard Institute, Queen Mary University of London, London, UK; 2 Northern Ireland Medical and Dental Training Agency (NIMDTA), Beechill House, 42 Beechill Rd, Belfast, BT8 7RL UK; 3 Zvitambo Institute for Maternal and Child Health Research, Harare, Zimbabwe; 4 Department of International Health, Johns Hopkins Bloomberg School of Public Health, Baltimore, MD USA

**Keywords:** Global health, Neonatal health, Neonatal mortality, Home delivery, Institutional delivery, Birth outcomes, Maternal health

## Abstract

**Background:**

Despite achieving relatively high rates of antenatal care, institutional delivery, and HIV antiretroviral therapy for women during pregnancy, neonatal mortality has remained stubbornly high in Zimbabwe. Clearer understanding of causal pathways is required to inform effective interventions.

**Methods:**

This study was a secondary analysis of data from the Sanitation Hygiene Infant Nutrition Efficacy (SHINE) trial, a cluster-randomized community-based trial among pregnant women and their infants, to examine care during institutional and non-institutional deliveries in rural Zimbabwe and associated birth outcomes.

**Results:**

Among 4423 pregnant women, 529 (11.9%) delivered outside a health institution; hygiene practices were poorer and interventions to minimise neonatal hypothermia less commonly utilised for these deliveries compared to institutional deliveries. Among 3441 infants born in institutions, 592 (17.2%) were preterm (< 37 weeks gestation), while 175/462 (37.9%) infants born outside health institutions were preterm (RR: 2.20 (1.92, 2.53). Similarly, rates of stillbirth [1.2% compared to 3.0% (RR:2.38, 1.36, 4.15)] and neonatal mortality [2.4% compared to 4.8% (RR: 2.01 1.31, 3.10)] were higher among infants born outside institutions. Among mothers delivering at home who reported their reason for having a home delivery, 221/293 (75%) reported that precipitous labor was the primary reason for not having an institutional delivery while 32 (11%), 34 (12%), and 9 (3%), respectively, reported distance to the clinic, financial constraints, and religious/personal preference.

**Conclusions:**

Preterm birth is common among all infants in rural Zimbabwe, and extremely high among infants born outside health institutions. Our findings indicate that premature onset of labor, rather than maternal choice, may be the reason for many non-institutional deliveries in low-resource settings, initiating a cascade of events resulting in a two-fold higher risk of stillbirth and neonatal mortality amongst children born outside health institutions. Interventions for primary prevention of preterm delivery will be crucial in reducing neonatal mortality in Zimbabwe.

**Trial registration:**

The trial is registered with ClinicalTrials.gov, number NCT01824940.

**Supplementary Information:**

The online version contains supplementary material available at 10.1186/s12884-022-05282-x.

## Introduction

Globally, neonatal mortality fell by 51% between 1990 and 2017 from 36.6 to 18.0 deaths per 1000 live births and the absolute number of annual neonatal deaths halved from 5 million to 2.5 million [1]. Despite these gains, more than 60 countries are not on track to meet the neonatal mortality (NNM) target of 12/1000 highlighted in the Sustainable Development Goals (SDGs) [1].

Most neonatal deaths occur during delivery [2, 3] or in the first 24 hours following birth [4, 5]. As such, efforts to reduce neonatal mortality have focused on encouraging and enabling women to deliver in health facilities, in the presence of skilled birth attendants (SBAs) [6, 7], which is associated with lower adverse outcomes in both infants and mothers [8–10].

Whilst many studies have focused on the reasons why women *choose* to deliver at home, there has been less discussion of women who intend to deliver in an institution but end up delivering at home when labour occurs unexpectedly early and /or progresses quickly. This situation may be especially relevant for women living in remote rural settings with poor infrastructure and limited transportation. Indeed in studies in Nepal, Kenya, and Tanzania examining reasons for non-institutional deliveries, one-third [11, 12] to two-thirds [13] of women reported precipitous or unexpectedly early labour as the primary reason they delivered at home.

In Zimbabwe, neonatal mortality has been a particularly stubborn problem: it increased from 27/1000 to 32/1000 live births between 1990 and 2019, in part reflecting economic hardship and high HIV prevalence [14]. This increase occurred despite high coverage of prevention of mother-to-child transmission (PMTCT) interventions (> 90% in 2018) [15], antenatal care (93% with ≥1 visit and 74% with ≥4 visits), and institutional deliveries (88%) [16]. The Sanitation Hygiene Infant Nutrition Efficacy (SHINE) trial was a cluster-randomized community-based trial conducted in two contiguous rural Zimbabwean districts (Chirumanzu and Shurugwi) which tested the independent and combined effects of improved infant and young child feeding (IYCF) and improved water, sanitation, and hygiene (WASH) on child health outcomes. This secondary data analysis from the SHINE trial provides an opportunity to examine the risk factors, birth practices and infant outcomes among women having institutional or non-institutional deliveries.

## Methods

### Sanitation hygiene infant nutrition efficacy (SHINE) trial

The SHINE trial has been previously described [17] and primary outcomes reported [18–20]. In brief, mothers and their infants were randomized to standard of care (SOC); IYCF (small-dose lipid-based nutrient supplement and complementary feeding counselling from 6 months of age); WASH (commencing during pregnancy with pit latrine and 2 hand-washing stations, liquid soap and chlorine, a clean play space, and hygiene counselling); or IYCF + WASH (all interventions). Primary outcomes were infant length and haemoglobin at 18 months, with several secondary outcomes, including child neurodevelopment, infant diarrhoea prevalence, incidence, and severity, and adverse birth outcomes.

### Data collection and analysis

From November 22nd, 2012, to March 27th, 2015, Village Health Workers (VHWs) employed by the Ministry of Health and Child Care (MoHCC) conducted prospective pregnancy surveillance by visiting all women aged 15–49 years in the study area to identify those who had missed a menstrual period and offering them a urine pregnancy test. New pregnancies were referred to research nurses who obtained written informed consent and enrolled women into the trial.

Home visits were carried out at baseline (~ 2 weeks after enrolment), at 32 gestational weeks, and at infant ages 1, 3, 6, 12 and 18 months to assess baseline characteristics and trial outcomes. Given the household nature of the WASH intervention, visits were only conducted when the mother was available in the home where she had been recruited, except at the 18-month visit (trial endpoint) when they were visited anywhere in Zimbabwe. Information about the delivery and the infant at birth was collated from the mother’s handheld records, the health facility records and by questionnaire at the 1-month postpartum visit or, for mother-infant dyads not available for the 1-month visit, at their first available postpartum visit. The trial provided Tanita BD-590 infant scales (Arlington Heights, IL, USA) to all institutions in the study area and trained health facility staff to use the scales and record infant birth weight on facility and patient-held records. Recumbent infant length was measured to the nearest 0.1 cm using a Seca 417 infantometer (Weigh & Measure LLC., Olney, MD, USA) by a research nurse during home visits at 1 month as an indicator of fetal linear growth. Gestational age at delivery was calculated from the date of the mother’s last menstrual period; values which were < 24 weeks or > 42 weeks + 6 days were excluded from analyses. Infants were classified as preterm (gestational age at delivery < 37 weeks), small-for-gestational-age (SGA; birthweight <10th percentile for gestational age using the INTERGROWTH reference [21]), or both preterm and SGA. Mean gestational age at delivery and proportion of infants born preterm (< 37 weeks) were estimated among two populations: first, only among infants with complete and plausible data, defined as those with birthweight-for-gestational age > 0.4th centile and < 99.6th centile using INTERGROWTH references (Estimate 1); and second, including infants in Estimate 1 plus infants whose birth weight was missing (Estimate 2). Infant length at 3 month was converted to Z-scores using the WHO reference [22].

Fetal losses and neonatal deaths were identified and reported to the trial by a research nurse, VHW, or the mother. Details of the event were reported by a research nurse to the study physician who reviewed the reports and classified the event as miscarriage (fetal loss before 28 weeks’ gestation), stillbirth (fetal loss after 28 weeks’ gestation), or neonatal death (live birth followed by death within the first 28 days) and reported them to the institutional review boards which approved and oversaw the trial (Medical Research Council of Zimbabwe and Johns Hopkins Bloomberg School of Public Health). Women gave written informed consent to participate.

### Statistical analysis

Baseline characteristics, care practices, and birth outcomes of women who had institutional compared to non-institutional deliveries were compared by calculating the mean difference (95% CI) for continuous variables and relative risk (95% CI) for categorical variables. All statistical analyses were performed using STATA version 14 [23]. Selection of care practices was guided by the WHO safe childbirth checklist (ref).

## Results

Five thousand, two hundred eighty pregnant women were enrolled from 211 clusters at a median gestational age of 12 (IQR 9–16) weeks (Supplementary Figure). During the antenatal period, 11 women were excluded, and one woman was added to the analysis to correct for enrolment errors; 139 women withdrew from the trial or were lost to follow-up, 4 died during pregnancy and 249 had a miscarriage. With the addition of 82 fetuses in twin/triplet pregnancies there were a total of 4956 fetuses delivered by 4878 mothers. Of these, place and details of the delivery was known for 4494 fetuses (90.7%) (4423 mothers); 3958 fetuses (88.1%) (3894 mothers) were delivered in an institution and 536 fetuses (11.9%) (529 mothers) were delivered outside a health institution.

Compared to women who delivered in a health institution, women who delivered outside a health institution were older, less likely to be primiparous, more likely to have been depressed during pregnancy, more likely to belong to the Apostolic faith and to have a lower socioeconomic status including fewer years of education, and poorer sanitation and drinking water quality (Table 1). Whilst mothers who had non-institutional deliveries were less likely to have had an HIV test prior to joining SHINE (RR 0.91, 95%CI 0.84–0.97), they were 39% more likely to test HIV-positive during the baseline visit of the trial. History of previous neonatal death, miscarriage, and stillbirth did not significantly vary by place of delivery.

**Table 1:** Characteristics of mothers and their household according to place of delivery

Delivery practice or condition	Place of delivery		Mean difference (95% CI); ***p*** value or RR (95% CI); ***p*** value
	Health Institution	Non-Institution	
	Mean (SD) [N] or No. (%) [N]	Mean (SD) [N] or No. (%) [N]	
Age, years	26.2 (6.7) [3462]	27.5 (6.7) [479]	+ 1.3 (0.6; 1.9); < 0.001
Height, cm	159.7 (8.6) [3795]	160.3 (8.6) 514]	+ 0.6 (−0.1; 1.4); 0.110
Mid-upper arm circumference, cm	26.4 (3.2) [3828]	26.0 (2.7) [519]	−0.4 (− 0.7; − 0.1); 0.007
Anaemic, Hb < 12 g/dl	1474 (37.2%) [3964]	202(37.8%) [534]	1.01 (0.91; 1.23); 0.826
Previously had had an HIV test	2480 (62.6%) [3964]	312 (58.4%) [534]	0.91 (0.84; 0.97); 0.006
Tested HIV-positive at trial enrolment	563 (14.2%) [3964]	106 (19.9) [534]	1.39 (1.15; 1.67); 0.001
Depressed ^a^	280 (7.1%) [3964]	52 (9.7%) [534]	1.36 (1.03; 1.80); 0.031
Education, years of schooling	9.6 (1.8) [3680]	9.1 (2.0) [506]	−0.5 (−0.7; − 0.3); < 0.001
Member of apostolic faith	1694 (42.7%) [3964]	298 (55.8%) [534]	1.27 (1.17; 1.38); < 0.001
Married	3481 (87.8%) [3964]	475 (89.0%) [534]	0.99 (0.97; 1.02); 0.580
Age at first marriage, years	19.1 (3.7) [2300]	18.6 (2.4) [287]	−0.51 (−0.94; − 0.07); 0.021
Mother is primiparous	537 (13.5%) [3964]	35 (6.6%) [534]	0.53 (0.39; 0.73); < 0.001
Had a previous neonatal death	106 (2.7%) [3964]	16 (3.0%) [534]	1.09 (0.65; 1.81); 0.741
Had a previous stillbirth	66 (1.7%) [3964]	8 (1.5%) [534]	1.00 (0.48; 2.06); 0.995
Had a previous miscarriage	181 (4.6%) [3964]	27 (5.1%) [534]	1.23 (0.83; 1.81); 0.297
Household size	4.9(2.2) [3732]	5.0(2.3) [509]	0.11 (−0.09; 0.32); 0.280
Has a household latrine	1349 (34.0%) [3964]	132 (24.7%) [534]	0.71 (0.61; 0.82); < 0.001
Uses improved source of drinking water	2290 (57.8%) [3964]	262 (49.1%) [534]	0.82 (0.76; 0.90); < 0.001
Food insecure (CSI > 10)^b^	639 (16.1%) [3964]	102 (19.1%) [534]	1.16 (0.96; 1.40); 0.119

^a^Depression defined as Edinburgh Postnatal Depression Scale (EPDS) score ≥ 12 and/or suicidal ideation which has been previously validated by psychometric testing among Zimbabwean women (Chibanda D et al.: Validation of the Edinburgh Postnatal Depression Scale among women in a high HIV prevalence area in urban Zimbabwe. *Arch Womens Ment Health* 2010, 13(3):201–206)

^b^*CSI *Coping Strategy Index (Maxwell D, Watkins B, Wheeler R, Collins G: The coping strategies index: A tool for rapidly measuring food security and the impact of food aid programs in emergencies. *Nairobi: CARE Eastern and Central Africa Regional Management Unit and the World Food Programme Vulnerability Assessment and Mapping Unit* 2003)

Many conditions and care practices during delivery differed between institutional and non-institutional deliveries (Table 2) [24]***.*** Women who delivered outside a health institution were less likely to have paid for delivery than those who delivered at a health institution. Only a small number (*N* = 25, 5.1%) of non-institutional deliveries were assisted by a healthcare professional (doctor, nurse, or midwife), compared to almost all (*N* = 3857, 99.5%) births at health institutions. Instead, non-institutional deliveries were more commonly assisted by VHWs, traditional birth attendants, faith healers, friends, or relatives. Several indicators suggested that fewer hygiene measures were taken during non-institutional births: birthing assistants were less likely to use gloves (RR 0.68, 95%CI 0.64–0.73), sterile blades to cut the cord (RR 0.97, 95%CI 0.93–1.00), or sterile string to tie the cord (RR 0.36, 95%CI 0.32–0.41). Unclean string was used to tie the cord in 22.5% (*N* = 101) of non-institutional births. Furthermore, infants born outside an institution were less likely to be dried (RR 0.72, 95%CI 0.66–0.79) and placed skin-to-skin with the mother (RR 0.22, 95%CI 0.18–0.28) before delivery of the placenta, which are both important indicators of neonatal hypothermia risk [25, 26]. Among 293 women who provided Information on their reason for having had a home delivery, 221 (75%) reported that precipitous labor was the primary reason for not having an institutional delivery while 32 (11%), 34 (12%), and 9 (3%), respectively, reported distance to the clinic, financial constraints, and religious/personal preference.

**Table 2:** Conditions and care practices during delivery according to place of delivery

	Place of Delivery						
**Condition or care practice**	**Institution**			**Home**			**RR (95% CI)**
	n	N	%	n	N	%	
Paid for delivery	1821	3828	47.6%	76	462	16.5%	0.35 (0.28; 0.43)
Person assisting with delivery							
*Doctor, nurse, or midwife*	3857	3878	99.5%	25	490	5.1%	0.05 (0.03; 0.07)
*VHW, TBA or Faith Healer*	3	3878	0.1%	74	490	15.1%	195.22 (61.78; 616.82)
*Friend, relative or other person*	53	3878	1.4%	399	490	81.4%	59.58 (45.45; 78.10)
*Traditional birth attendant*	0	3878	0.0%	54	490	11.0%	–
Birthing assistant wore gloves	3630	3661	99.2%	338	490	69.0%	0.68 (0.64; 0.73)
Used plastic sheet	3540	3732	94.9%	394	502	78.5%	0.83 (0.79; 0.87)
Delivered early for medical indication	108	3831	2.9%	7	432	1.6%	0.57 (0.27; 1.23)
When was cord cut relative to placenta delivery							
*Before placenta*	2142	2716	78.9%	190	440	43.2%	0.55 (0.49; 0.61)
*After placenta*	574	2716	21.1%	250	440	56.8%	2.69 (2.41; 3.00)
Instrument used to cut cord							
*Sterile blade*	2504	2730	91.7%	393	444	88.5%	0.97 (0.93; 1.00)
*Boiled blade*	17	2730	0.6%	10	444	2.3%	3.62 (1.67; 7.85)
*Washed blade*	3	2730	0.1%	15	444	3.4%	30.7 4(8.9; 105.74)
*Unwashed blade*	0	2730	0.0%	10	444	2.3%	–
*Other*	206	2730	7.6%	16	444	3.6%	0.48 (0.29; 0.79)
Anything applied to cord immediately after cutting							
*Yes*	372	2697	13.8%	60	442	13.6%	0.98 (0.76; 1.27)
*No*	2325	2697	86.2%	382	442	86.4%	1.00 (0.96; 1.04)
Used to tie the cord?							
*Sterile string*	3211	3384	94.9%	155	450	34.4%	0.36 (0.32; 0.41)
*Boiled string*	16	3384	0.5%	15	450	3.3%	7.05 (3.51; 14.16)
*Clean string*	71	3384	2.1%	172	450	38.2%	18.22 (14.07; 23.59)
*Unclean string*	3	3384	0.1%	101	450	22.4%	253.17 (80.64; 794.83)
*Other*	83	3384	2.5%	7	450	1.6%	0.63 (0.30; 1.36)
Baby dried before placenta delivered	2262	2909	77.8%	242	432	56.0%	0.72 (0.66; 0.79)
Baby washed with water before placenta							
*Yes*	111	3193	3.5%	19	447	4.3%	1.22 (0.76; 1.97)
*No*	3082	3193	96.5%	428	447	95.8%	0.99 (0.97; 1.01)
Baby placed skin-to-skin before placenta delivered							
*Yes*	2091	3220	64.9%	65	446	14.6%	0.22 (0.18; 0.28)
*No*	1129	3220	35.1%	381	446	85.4%	2.44 (2.29; 2.59)

Infants with non-institutional deliveries were more likely to have low birthweight (RR 1.65, 95%CI 1.24–2.19) (< 2.5 kg) and more than 3 times (95%CI 1.48–7.77) as likely to have very low birth weight (< 1.5Kg) (Table 3). Among infants born in institutions, 592/3441 (17.2%) were preterm (< 37 weeks gestation), while 175/462 (37.9%) of infants born outside health institutions were preterm (RR: 2.20 (1.92, 2.53) (Table 3, Estimate 2). Rates were slightly attenuated when infants who did not provide birthweight were excluded 555/3288 (16.9%) and 82/253 (32.4%) (Table 3, Estimate 1). Similarly, rates of stillbirth [1.2% compared to 3.0% (RR:2.38, 1.36, 4.15)] and neonatal mortality [2.4% compared to 4.8% (RR: 2.01 1.31, 3.10)] were higher among infants born outside compared to inside health institutions.

**Table 3:** Infant birth outcomes by place of delivery

Infant birth outcome	Place of Delivery			
	Institution	Non-Institution		Birthplace Unknown
	No. (%) [N] or Mean (SD) [N]	No. (%) [N] or Mean (SD) [N]	RR (95% CI) p or Mean diff. (95% CI) p	No. (%) [N] or Mean (SD) [N]
**Female**	1961 (49.3%) [3979]	262 (49.8%) [526]	1.02 (0.93, 1.11); 0.733	164 (48.5%) [338]
**Birth weight (kg)**	3.1 (0.5) [3824]	2.9 (0.6) [316]	−0.17(−0.23, −0.11); < 0.001	3.0 (0.5) [161]
**Low birthweight**				
**< 2.5 Kg**	344 (9.0%) [3824]	47 (14.9%) [316]	1.65 (1.24, 2.19); < 0.001	3 (1.9%) [161]
**< 2.0 Kg**	94 (2.5%) [3824]	24 (7.6%) [316]	3.09(2.00; 4.77)0.000	3 (1.9%) [161]
**< 1.5 Kg**	25 (0.7%) [3824]	7 (2.2%) [316]	3.39(1.48;7.77)0.004	3 (1.9%) [161]
**Gestational age at delivery (weeks)**				
*Estimate 1*	38.9 (2.4) [3288]	37.7 (3.0) [253]	−1.15 (−1.46, −0.84); < 0.001	38.4 (2.8) [126]
*Estimate 2*	38.8 (2.6) [3440]	37.3 (4.4) [462]	−1.54 (−1.81, −1.26); < 0.001	38.2 (4.4) [295]
**Preterm (< 37 wk)**				
*Estimate 1*	555 (16.9%) [3288]	82 (32.4%) [253]	1.92 (1.58, 2.33); < 0.001	29 (23.0%) [126]
*Estimate 2*	592 (17.2%) [3441]	175 (37.9%) [462]	2.20 (1.92, 2.53); < 0.001	85 (28.1%) [303]
**Small-for-gestational age (<10th centile)**	524 (15.9%) [3288]	44 (17.4%) [253]	1.09 (0.82, 1.44); 0.541	23 (18.3%) [126]
**Preterm AND Small-for-gestational age**	33(1.0%) [3288]	8(3.2%) [253]	3.15 (1.47, 6.75); 0.003	2(1.6%) [126]
**Stillbirth**	50 (1.2%) [4029]	16 (3.0%) [542]	2.38 (1.36, 4.15); 0.002	47 (12.2%) [385]
**Neonatal Death**	94 (2.4%) [3979]	25 (4.8%) [526]	2.01 (1.31, 3.10); 0.001	33 (9.76%) [338]

*Estimate 1 – does not include those with missing birthweight*

*Estimate 2 – includes those with missing birthweight*

## Discussion

In the SHINE study population, 18.2% of infants were born preterm and 57% of both the neonatal deaths and stillbirths were among infants born prematurely [27]. This preterm birth rate is among the highest in the world. A key insight of the current analysis is that the proportion of infants born preterm was 2.2 (95% CI: 1.92, 2.53) times higher among infants with non-institutional compared to institutional deliveries (37.9% vs 17.2%). While previous studies have focussed on determinants of non-institutional deliveries which then lead to poorer birth outcomes [28–30], our observations imply the reverse: the highly prevalent (and unexpectedly early) preterm labor experienced by SHINE mothers may be the reason many of these mothers delivered outside a health institution. Moreover, we observed many of the same risk factors of non-institutional delivery (e.g., lower socioeconomic status) that have been reported by others. This implies that among the many women in Zimbabwe who experience preterm labor, those who are also poorer, less educated, and more depressed, lack the means to reach a health institution quickly, and so are attended by untrained caregivers in less hygienic conditions. This cascade of events likely contributed to the two-fold higher risk of stillbirth and neonatal mortality among non-institutional deliveries in our study. This offers a potential explanation for the findings of a recent study carried out in Zimbabwe which found that women, burdened by multiple interacting vulnerabilities related to poverty, were most likely to deliver ‘on the road’ whilst attempting to reach a healthcare institution [31].

In recent years, substantial progress has been made in scaling up interventions for small and sick neonates. These include affordable devices for continuous positive airway pressure for respiratory distress syndrome [32, 33], training health workers in neonatal resuscitation [34], surfactant therapy for premature infants [35] and steroid [36] and antibiotic [37] therapy for meconium aspiration and severe infection. While these interventions have made huge contributions to improving neonatal survival, all are hospital-based. Our observation that at least 20% of the preterm births in the SHINE study population occurred outside a health institution, demonstrates that in addition to interventions for enhanced neonatal care, there is an urgent need for interventions that prevent preterm labor. There are now three evidence-based interventions for preventing preterm birth which are low cost and safe during pregnancy. In populations with low dietary calcium intake, antenatal calcium supplementation at doses of ≥1 g per day can reduce preterm birth by 24% according to a recent Cochrane Review [38]. Indeed, since 2016, the World Health Organization has recommended 1.5–2.0 g calcium supplementation throughout pregnancy for women with low dietary calcium primarily for its effect on reducing preeclampsia, although this recommendation has not been widely scaled up. In a recent trial among 12,000 pregnant women in 6 LMICs, daily low-dose (81 mg) aspirin reduced preterm birth by 11% [39] without any excess adverse side effects. Replacing iron-folate with multiple micronutrient supplementation may also modestly reduce the risk of preterm birth, [40] especially when initiated early in pregnancy [41]. Other interventions which might be considered in the future include omega-3-poly-unsaturated fatty acids (shown to reduce preterm birth in most [42, 43] but not all [44] trials) and anti-inflammatory drugs (e.g., a trial of cotrimoxazole, which has potent anti-inflammatory effects [45], is underway in Zimbabwe (PACTR202107707978619) and pharmaceutical preparations of specialized proresolving lipid mediators are under development [46, 47]).

## Conclusion

This study supports the existing literature in describing the sociodemographic profiles of women who have non-institutional deliveries in rural Zimbabwe. These women are often poorer, less well educated, and more likely to have HIV than those women who give birth at a health institution. As would be expected, the standard of care which women receive outside a health institution is inferior to that provided in health institutions, with poorer access to experienced health professionals and sanitation.

Our findings indicate that preterm birth rates are particularly high amongst non-institutional deliveries, suggesting that premature onset of labor, rather than maternal choice, may be the reason for many home deliveries. Interventions for primary prevention of preterm delivery will be crucial in reducing neonatal mortality in Zimbabwe.

## Supplementary Information

**Additional file 1: Supplementary Figure.** Participant flow for analyses examining antenatal and delivery practices among non-institutional and to institutional deliveries.

## Data Availability

The datasets used and/or analysed during the current study are available from the corresponding author on reasonable request.

## Abbreviations

NNM: Neonatal mortality
SDGs: Sustainable Development Goals
SBAs: Skilled Birth Attendants
PMTCT: Prevention of mother-to-child transmission
SHINE: Sanitation Hygiene Infant Nutrition Efficacy
IYCF: Infant and young child feeding
WASH: Water, sanitation, and hygiene
SOC: Standard of care
VHWs: Village health workers
MoHCC: Ministry of Health and Child Care
SGA: Small for gestational age
WHO: World Health Organisation
HIV: Human Immunodeficiency Virus
LMICs: Low- and middle-income countries
