# Supplementary material for: Antenatal and delivery practices and neonatal mortality amongst women with institutional and non-institutional deliveries in rural Zimbabwe: observational data from a cluster randomized trial
Source: BMC Pregnancy Childbirth. 2022 Dec 30;22:981. doi: 10.1186/s12884-022-05282-x (PMC9805263; doi:10.1186/s12884-022-05282-x)
Supplement: Supplementary file 1 — Additional file 1: Supplementary Figure. Participant flow for analyses examining antenatal and delivery practices among non-institutional and to institutional deliveries. [file 12884_2022_5282_MOESM1_ESM.docx]

**Supplementary Figure**. Participant flow for analyses examining antenatal and delivery practices among non-institutional and to institutional deliveries.

Consented Mothers

5280

1 Added to correct for enrolment errors

11 Enrolled by error

Enrolled Mothers

5270

462 fetuses (455 mothers) place of and/or care practices at delivery missing

249 Miscarriages

4 Maternal deaths

90 Lost to follow-up

**536 fetuses (529 mother)**

**Home Deliveries**

**3958 fetuses (3894 mothers)**

**Institutional Delivery**

82 Additional fetuses in twin/triplet pregnancies

4956 fetuses delivered by 4878 mothers

49 Exited mothers
